# Biomechanical limits of hopping in the hindlimbs of giant extinct kangaroos

**DOI:** 10.1038/s41598-025-29939-7

**Published:** 2026-01-22

**Authors:** Megan E. Jones, Katrina Jones, Robert L. Nudds

**Affiliations:** 1 Department of Earth and Environmental Sciences, The University of Manchester, Williamson Building, Oxford Road, Manchester, M13 9PL UK; 2 School of BioSciences, The University of Melbourne, Grattan Street, Parkville, VIC 3010 Australia; 3 Present Address: School of Earth Sciences, City of Bristol, The University of Bristol, Wills Memorial Building, Queens Road, Bristol, BS8 1RJ UK; 4 School of Biological Sciences, Faculty of Biology, Medicine and Health, The University of Manchester, Oxford Rd, M13 9PL Manchester, UK

**Keywords:** Kangaroo, Hindlimb, Biomechanics, Hopping, Sthenurine, Protemnodon, Anatomy, Ecology, Ecology, Evolution, Zoology

## Abstract

The locomotor abilities of animals depend upon their body size. Today, kangaroos are the largest hopping mammals, but some of their Pleistocene relatives were larger still—more than twice as heavy as any modern kangaroo. So, is there an upper size limit of bipedal hopping? Previous analyses have recovered an upper limit of ~ 140–160 kg based on allometry, but have suggested that incorporating changes in hindlimb scaling patterns among giant species would alter these conclusions. Here, we test this proposal by integrating scaling data from modern kangaroos with direct observation of the hindlimb bones of giant fossil kangaroos. We test two potential limiting factors on hopping—bone strength, and tendon size. We find that (a) the metatarsals of giant kangaroos would be capable of resisting the bending moments involved in hopping, and (b), the calcanea (heel bones) of giant kangaroos could accommodate tendons large enough to resist the loads generated during hopping. While hopping may not have been their primary mode of locomotion, our findings suggest that it may have formed part of a broader locomotor repertoire, for example for short bursts of speed.

**Supplementary Information:**

The online version contains supplementary material available at 10.1038/s41598-025-29939-7.

## Introduction

Body mass has a profound impact on animal locomotion^1–3^. Many mammals compensate for increasing loads associated with larger sizes by adopting an increasingly upright stance, which minimises force by reducing the lever arm of the ground reaction forces around the limb joints^4^. However, this is not possible in bipedal hopping mammals, because hopping requires a crouched posture, so we might expect the upper body mass limit for hopping to be lower than the limit for similarly energetic quadrupedal gaits. Bipedal hopping has evolved independently in only five extant lineages^5^,of which only the Macropodiformes (kangaroos, wallabies and their relatives) have reached body masses far above 3 kg^5,6^ The largest members of the group today (~ 90 kg, male *Osphranter rufus*^5,7^ are capable of hopping; but a variety of Pleistocene macropodoids were much larger, with some reaching masses of up to 250 kg (Helgen et al., 2006). Were these giant extinct species too large to hop^8–10^?

**Fig. 1:**
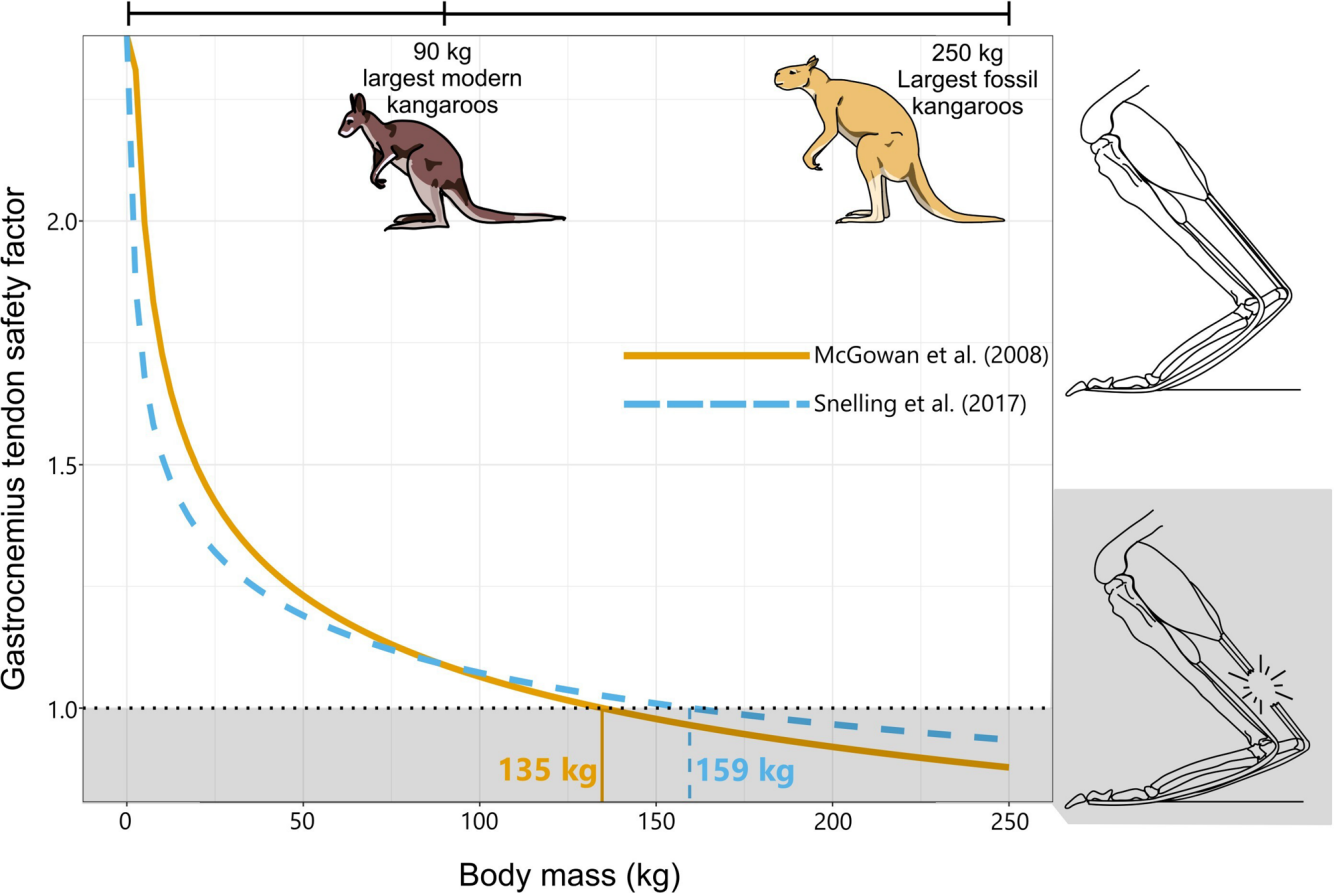
Illustration of previous studies’ results suggesting a size limit of 135–160 kg for hopping in giant kangaroos, based on the scaling patterns of the gastrocnemius tendon safety factor among modern kangaroos. Both curves (Solid^10^; Dashed^11^) are based only on data from modern kangaroos. Safety factors below one indicate tendon rupture. Labelled vertical lines indicate the mass at which each allometric curve predicts safety factor to drop below one. Illustrations by MJ. Hindlimb image based on^10,25,59^.

Giant extinct macropodiforms share the general body plan of their smaller hopping relatives, but previous work suggests that their hindlimbs would not have been able to withstand the forces involved in hopping. The best estimates so far have placed the body mass limit for hopping at approximately 135–160 kg^10,11^, a mass that several giant kangaroo lineages exceed (Fig. 1). However, these studies derive their estimates by extrapolating the allometric scaling pattern of living kangaroos. They use the ankle tendon morphology of modern kangaroos to predict the mass at which the safety factor (the ratio of the failure stress of a structure to the maximum stress experienced by that structure) of the tendons would drop below one, indicating rupture^10–12^. Extrapolating allometry beyond the limits of the modern data is problematic because it assumes the same scaling patterns of the ankle extensor tendons in giant extinct kangaroo species as in smaller macropodoids. Both studies acknowledge these limitations, and suggest that the giant kangaroos may still have been able to hop, either through altered scaling patterns, such as possessing relatively thicker tendons, or through hopping at reduced speeds to lower the stresses involved. Incorporating evidence directly from the fossil record allows these suggestions to be tested, as it can provide more accurate estimates of scaling relationships. Previously, an abstract by McGowan^13^ reported that giant kangaroos do indeed possess thicker ankle extensor tendons than expected based on allometry, making hopping more plausible in these species. Here, we investigate further, providing the first full study to empirically test these previous suggestions.

**Fig. 2:**
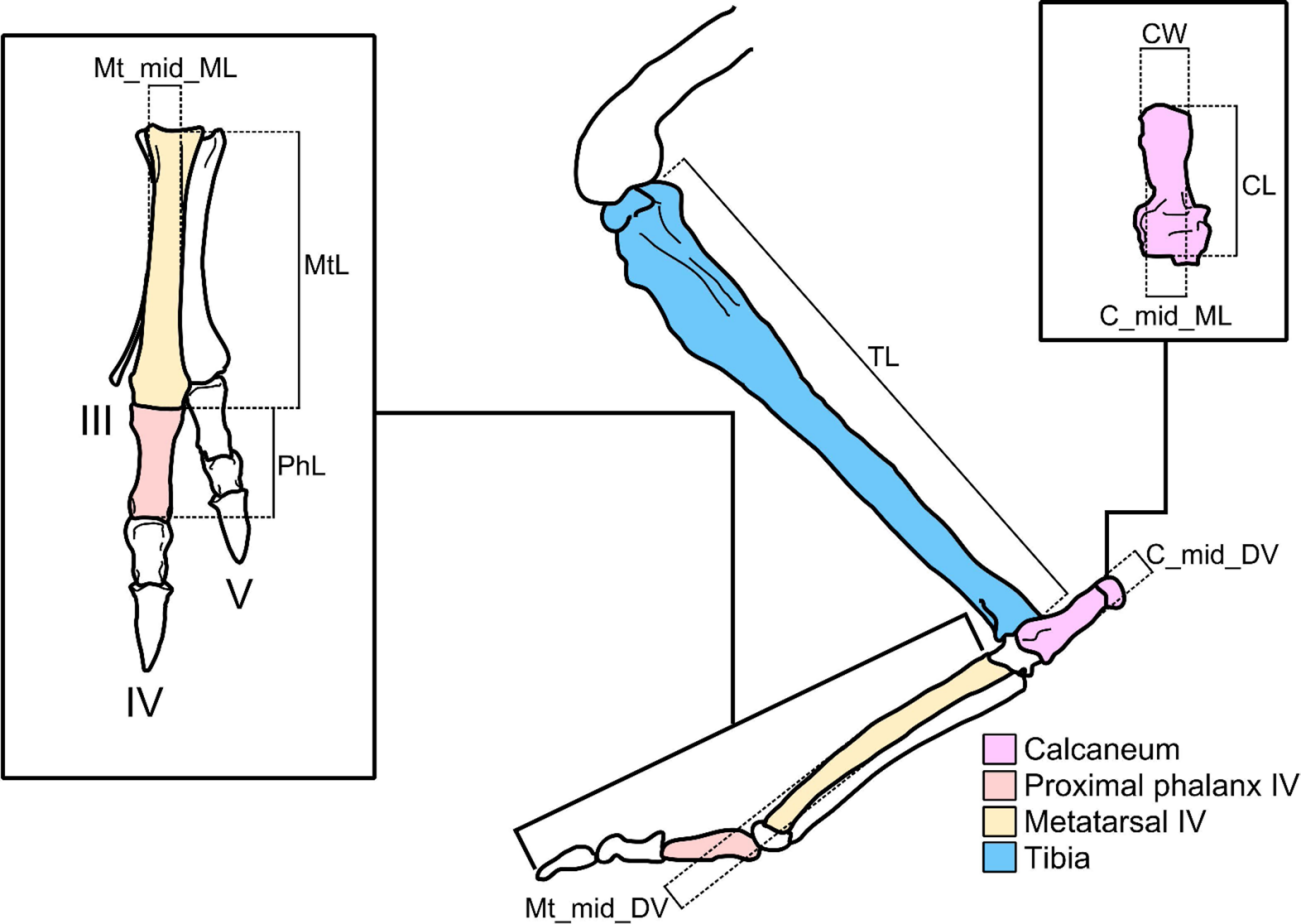
Illustration of a representative kangaroo hindlimb, with key bones and length measurements referred to in this study highlighted. Inserts show a dorsal view of the foot and calcaneum respectively. Abbreviations: C_mid_DV = calcaneum midshaft mediolateral width; CL = calcaneum length; C_mid_ML = calcaneum midshaft dorsoventral width; CW = calcaneal tuberosity width; Mt_mid_DV = metatarsal IV midshaft dorsoventral width; Mt_L = metatarsal IV length; Mt_mid_ML = metatarsal IV midshaft mediolateral width; PhL = proximal phalanx IV length; TL = tibia length. Illustrations by MJ; hindlimb image as per Fig. 1, with insert illustrations based on photograph courtesy of Christine Janis.

To estimate the feasibility of hopping in giant kangaroos, we investigated the strength of the hindlimb bones, and the capability of ankle extensor tendons in resisting hopping loads. We test two hypotheses, both of which must be supported for hopping to be plausible in these species. (1) Metatarsal IV safety factors will not drop below one when hopping. Previous studies of hopping-related stresses in hindlimb bones focus primarily on the tibia^14–16^. However, the metatarsals are the least robust of the hindlimb long bones (having the smallest diameter) and will, therefore, experience the greatest bending moments relative to total stress. Bone is most likely to break under bending loads^17^. Hence, if the metatarsals are unlikely to fracture due to bending under hopping forces, then other hindlimb bones are likely not at risk of fracture either. This is supported by the observation that, in thoroughbred racehorses—another cursorial mammal with reduced metatarsals/metacarpals—fractures in the third metacarpal were the most common^18^. Among the kangaroos, weight is borne on the fourth metatarsal, and to a varying degree, on the fifth, with the rest being reduced (Fig. 2). The Sthenurines in particular bear all of their weight on metatarsal IV. For the sake of simplicity and consistency, only the fourth metatarsal is tested in this study. (2) The ankle is robust enough to support the tendons required for hopping. Specifically, the insertion area for the gastrocnemius tendon (main ankle extensor for hopping) on the calcaneal tuberosity (insertion point of the gastrocnemius at the ankle) will be large enough to accommodate tendons that could resist the forces required for hopping. To test this hypothesis, we measured the width of the calcaneal tuberosity and compared it to three different estimates of the width of the gastrocnemius tendon.

## Results

### Hypothesis 1: metatarsal IV strength

**Fig. 3:**
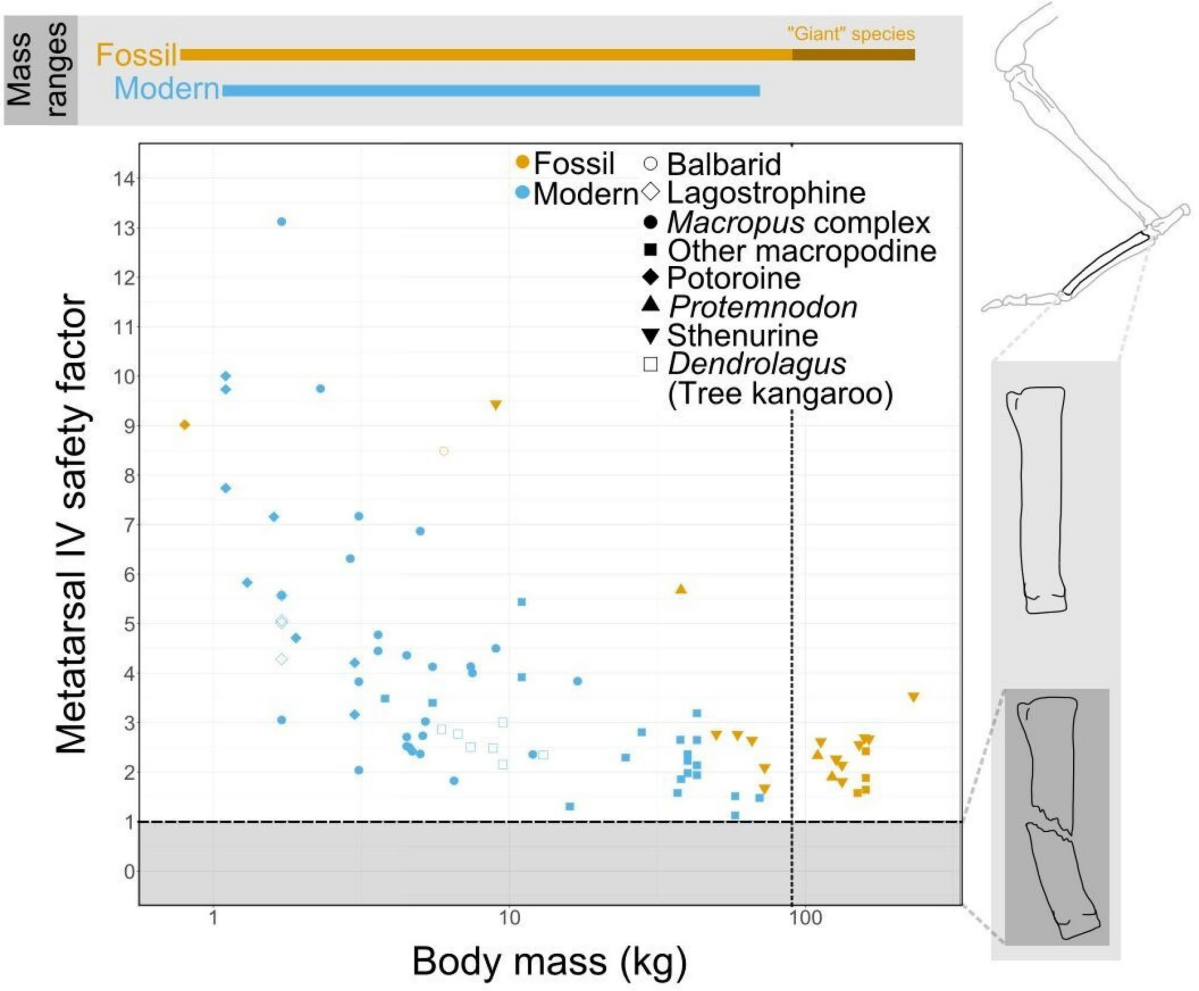
Scatterplot of the predicted safety factor of metatarsal IV at midstance when hopping, against log-transformed body mass. The shapes of the points denote clades within the Macropodoidea, while colour denotes whether an individual is modern or a fossil. The horizontal dashed line indicates a safety factor of one, below which the bone would be expected to fracture. The vertical dotted line indicates the mass of the largest known extant kangaroos (90 kg). Labelled mass ranges are for this dataset, not all known species. *n* = 89 individuals. Metatarsal outlines created by MJ. Hindlimb outline by MJ, based on^10,25,59^.

Hypothesis one posits that the hindlimb bones of the giant extinct species must be able to withstand the stresses hopping will subject them to without fracturing. The lowest predicted safety factor (1.12) for the fourth metatarsals is seen in the red kangaroo (*Osphranter rufus*) weighing 57.9 kg (Fig. 3). Among the giant extinct species, all individuals (including the sthenurines *Sthenurus stirlingi*, *Sthenurus tindalei*,* Simosthenurus occidentalis*, and an unclassified *Procoptodon* species, likely *P. goliah* based on similarity to the identified *P. goliah* in the dataset; *Protemnodon anak* and *P. viator*—AMNH FM145501, previously *P. brehus*^19^—and giant *Macropus* species *M. titan* and *M. ferragus*) were predicted to have similar safety factors, ranging from around 1.5 to 3.5: higher than those of many of the largest living species. This may indicate an adaptation to resist greater loads or may be a by-product of a reduced length of the metatarsals (Fig. S1).

### Hypothesis 2: ankle tendon size

Hypothesis two posits that, to permit hopping, the calcaneal tuberosity of the extinct giant species must be large enough to accommodate a tendon that is wide enough to transmit the muscle forces during hopping locomotion.

**Fig. 4:**
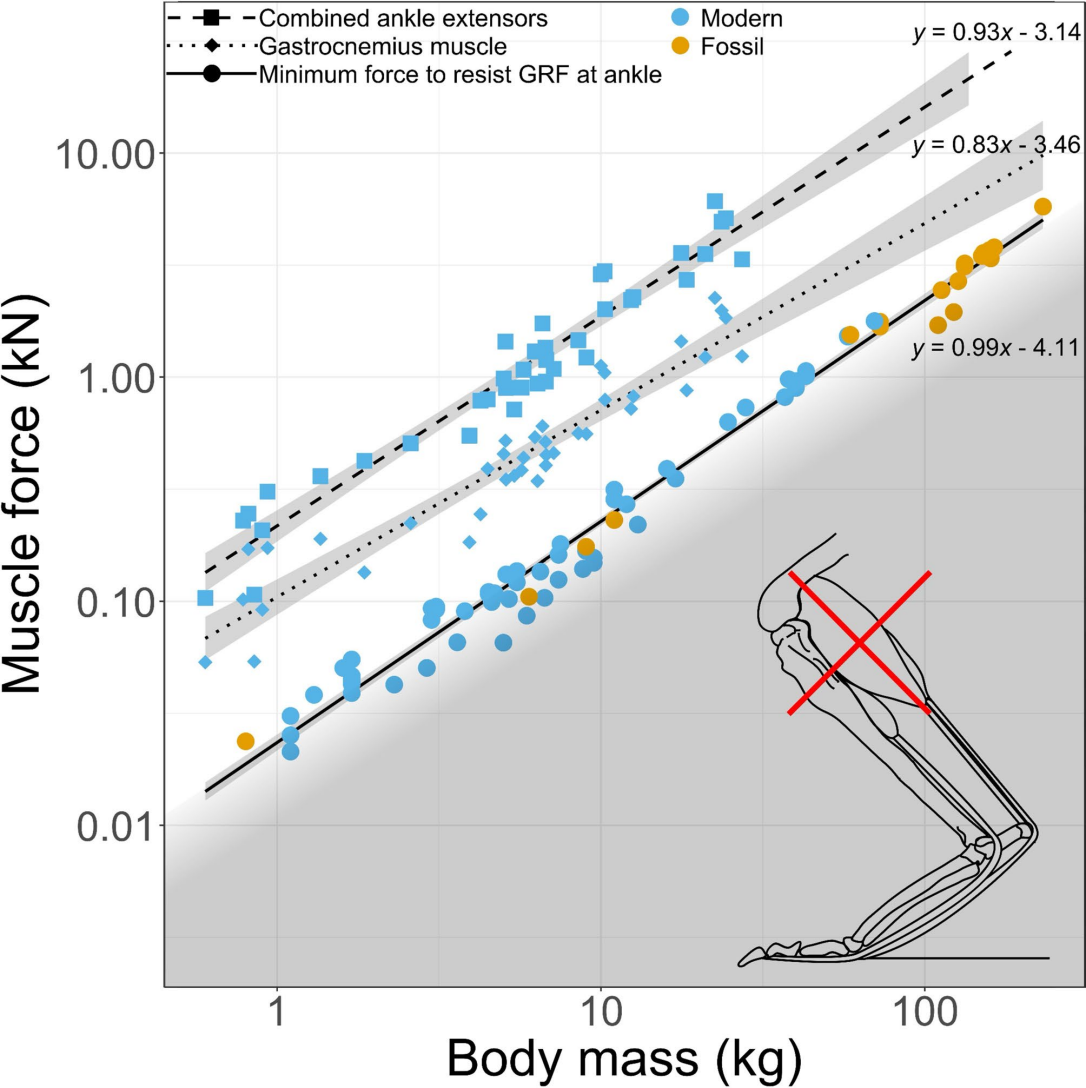
Muscle force plotted against body mass (*M*_b_). Muscle force is derived from three different methods of determining ankle extensor physiological cross-sectional areas (PCSAs), with predicted minimum PCSA based on ankle moments. Force is then derived from PCSA by multiplying by 300 (as a maximal isometric stress of 300 kPa is assumed, following^10^). Lines show linear least squares regressions, with 95% confidence intervals shaded, and labelled with their equations, where *y* is log_10_(PCSA), and *x* is log_10_(M_b_). Linear regression values are as follows. Total measured PCSA (dashed line): PCSA ∝ *M*_b_^0.935±0.081^ (P-value: <2e-16); measured gastrocnemius PCSA (dotted line): PCSA ∝ *M*_b_^0.833±0.091^(P-value: <2e-16); predicted from ankle moments (solid line): PCSA ∝ *M*_b_^0.986±0.027^(P-value:<2e-16). Shading below the lowest regression line indicates the approximate area in which muscles would be unable to resist ground reaction forces (circular points indicate actual limit for each individual). Note the log scale on each axis. *n* = 80 for the predicted PCSA from ankle moments; *n* = 39 for the measured PCSAs. Hindlimb image by MJ, based on^10,25,59^.

To test this, the relevant muscle forces must first be calculated. Physiological cross-sectional area (PCSA) is a proxy for the forces that can be produced by muscles The predicted minimum required ankle extensor muscle PCSA—calculated based on the minimum force needed to counteract the moment produced around the ankle by ground reaction forces while hopping—was consistently lower than the measured total ankle extensor muscle PCSA, and the predicted PCSAs for giant species based on those measurements (Fig. 4). The slopes of the total ankle extensor muscle PCSA and the predicted minimum PCSA were not significantly different from one another, and both scaled with hyperallometry. This scaling suggests that, among modern kangaroos, muscles scale at a rate proportional to increases in ground reaction force with body mass. As expected, muscles increase in size at an appropriate rate to accommodate increased forces associated with body mass.

**Fig. 5:**
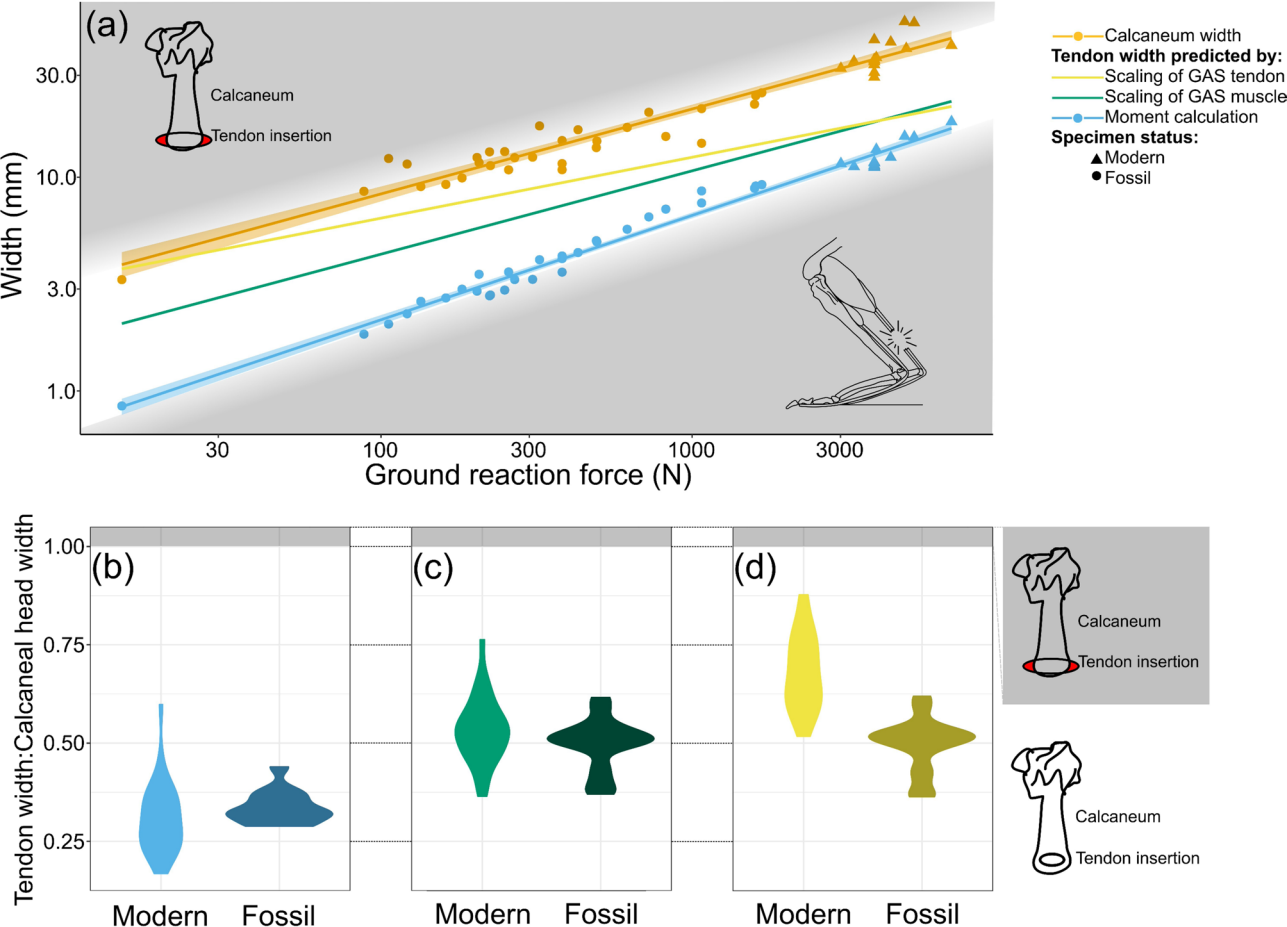
(**a**) Predicted gastrocnemius tendon widths, compared against actual calcaneal tuberosity widths. Shading around regression lines indicates 95% confidence intervals. Grey highlighting indicates approximate regions of implausible hopping, either due to tendons being too narrow to resist ground reaction forces (lower region), or due to the tendons being wider than the available insertion space (upper region). (**b-d**) Violin plots of ratio of predicted tendon width to calcaneal tuberosity width, in modern vs. fossil individuals, with region indicating tendons larger than available insertion area highlighted in grey. Tendon widths predicted based on (**b**) moment calculations, (**c**) scaling of gastrocnemius muscle, and (**d**) scaling of gastrocnemius tendon. *n* = 46; *n* = 43 for the moment-based calculation of tendon width. Calcaneum outline by MJ; Hindlimb image by MJ, based on^10,25,59^.

Using these PCSA estimates, we then tested if the calcaneum could accommodate a gastrocnemius tendon large enough to resist the forces generated by the gastrocnemius muscle during hopping. All three methods of predicting the diameter of the gastrocnemius tendon (the tendon inserting on the calcaneum) produced diameters smaller than the measured calcaneal tuberosity widths for the same individual (Fig. 5). Across the three methods, no fossil kangaroo showed higher ratios of predicted tendon width to measured calcaneal width than those of the modern individuals, while many showed a lower ratio than the largest modern kangaroos, suggesting relatively more robust calcanea than required for these tendons. This implies that hopping was mechanically possible for these species, and that they may have possessed more robust gastrocnemius tendons than expected, relative to body size.

## Discussion

Here we tested the hypotheses that hopping in extinct giant kangaroos may have been limited by (a) metatarsal IV bone strength or (b) strength of the ankle extensor (gastrocnemius) tendon. The results of this study suggest neither metatarsal nor gastrocnemius strength would prevent the giant kangaroos from hopping, challenging previous findings, based on tendon scaling, that this gait would have been mechanically impossible in the largest species, but supporting previous suggestions that an increased tendon width might ensure hopping remains possible in these species.

### Assumptions

In order to model the stresses and necessary muscle and tendon dimensions analysed in this study, several assumptions must be made about the mechanics of hopping in the kangaroos. First, we assume a constant posture when hopping in all species, using the measured joint angles in a medium-sized wallaby. Second, we assume that the ground reaction force (GRF) acts at the metatarsophalangeal joint. Both of these assumptions were tested, and supported, by sensitivity analyses (Fig. S2, S3). Finally, we assume that peak GRF is equal to three times body weight, and that it acts vertically. Both of these assumptions are supported in the literature, with the former assumption allowing for a comparison of adaptations assuming a comparable hopping speed and duty factor across species. These are variables that may very well be altered in different species, and where relevant, the effects of changes to these variables will be discussed. For details on each of these assumptions, and their justification, see methods.

### Metatarsal safety factors

We estimated the safety factors of fourth metatarsals, the least robust and most vulnerable hindlimb bones, in giant kangaroos. In the modern kangaroos, we see an apparent negative correlation between bone safety factor and mass, corresponding with previous observations of kangaroo tibiae^14–16^. While the relatively small red-necked wallaby (*Notamacropus rufogriseus*) has tibial stresses and safety factors within the range expected for an equivalent-sized quadruped, larger species such as *Osphranter rufus* experience unusually low tibial safety factors, outside the 2–4 range occupied by most mammals^14–16^. However, when fossil species are included, our results suggest that none of the giant kangaroos examined would have metatarsal safety factors below one, if they were to hop as their living relatives do. Thus, it seems unlikely that hindlimb bone strength would have been a limiting factor in the ability of giant kangaroos to hop. The consistency in bone safety factors in other mammals is produced by changes in stance which affect effective mechanical advantage^4,20,21^, rather than morphological changes to the hindlimb bones themselves. By contrast, our calculations assume a constant, crouched stance, but still find a levelling-off of metatarsal safety factors in the giant species. The relatively constant safety factors of the giant kangaroos must therefore be attributed to increasing robustness of the metatarsals. Indeed, they possess short metatarsals relative to the rest of the limb (Fig. S1). Shortening the metatarsals does reduce strain and thus increase the safety factor of the bone, as seen in other relatively short-footed species, such as the tree kangaroos (Fig. S1). However, the trade-off for possessing a shorter, stronger metatarsal is a reduced out-lever of the ankle extensors, and a consequent reduction in take-off (hopping) speed. Thus, although the limb bones of giant kangaroos may be robust, there is a likely trade off with acceleration.

This study calculates metatarsal bone strength from external geometry, disregarding internal geometry. While internal geometry can impact bone strength, and a previous study of the internal geometry of giant kangaroo pedal bones has revealed significant differences between lineages^22^, external geometry has been used extensively to characterise bone strength in mammals, e.g^1,16^. and has the greatest overall impact on strength. Therefore, a study of internal geometry, which would involve data from radiographs, is beyond the scope of the study. Given that our findings for the living kangaroos in this study align well with previous findings studying the in vivo stresses of kangaroo hindlimb long bones^14–16^, our methods can be assumed to produce a reasonable approximate measure for bone strength.

One factor not accounted for in our calculations is the differing number of metatarsals bearing weight across the kangaroos. In the sthenurines, weight is borne only on the fourth metatarsal, while in all other kangaroos—including other giant species—weight is borne on both the fourth and fifth metatarsals, with the fifth metatarsal being particularly robust in *Protemnodon*^19^ This does mean that our results may underestimate the safety factors of the fourth metatarsal in all non-sthenurines, as for the sake of simplicity and the available data, our calculations assume weight is borne only on the fourth metatarsal. However, an increased safety factor for non-sthenurines would not affect our ultimate conclusion for this first hypothesis. It is worth noting, though, that *Protemnodon anak* is here recovered as having a similar metatarsal safety factor to similarly-sized sthenurines. If the robust fifth metatarsal of this species were to be included, this safety factor would likely be significantly higher than that experienced by the sthenurines, suggesting that *P. anak* possesses an unusually robust pes.

### Ankle extensor tendon insertion area

Next, we tested the ability of calcanea to accommodate the extensor (gastrocnemius) tendons required for hopping. Our results indicate that there would have been adequate space for the insertion of even the largest tendons from our three tendon width estimation methods. This remains true whether the estimates derive from allometric extrapolation, or purely biomechanical calculations from body mass and limb bone lengths. This contradicts previous results suggesting that the gastrocnemius tendon would be insufficient to support hopping in giant kangaroos based on tendon scaling in modern kangaroos, but confirms the suggestions of previous studies^10,11,13^ that increased tendon width in giant species above allometric predictions might still allow for hopping. Indeed, the most likely factor driving the difference between our conclusions and the findings of these prior studies, is the relatively shorter and broader calcanea of the giant kangaroos compared to living species, indicating the potential for more robust tendons than would be assumed based on extant scaling alone (Fig. S4).

Calcaneum length scales with hypoallometry relative to body mass in sthenurines^23^, decreasing the in-lever of the ankle extensor muscles, which would increase the muscle force necessary to resist ground reaction forces. As an adaptation, this might be expected to limit hopping. However, our calculations take the length of the calcaneum into account, and still find that the calcanea are capable of accommodating the required tendons. We do not here consider the available insertion area for the plantaris tendon (another ankle extensor); while not as key as the gastrocnemius, this aspect may require further investigation.

It is also worth noting that the calculations in this study are conservative in that they assume a hopping speed equivalent to that seen in modern kangaroos. It is entirely possible that, as well as using hopping more infrequently, or over shorter distances, the giant kangaroos may have reduced stresses by hopping more slowly. While our results do not indicate that this would have been necessary for any of the species in this study, it is a possibility that must be taken into consideration before ruling hopping infeasible in any giant species.

The calculations in this study do assume that the maximum stress exerted by a muscle is its maximum isometric stress. This may very well not be accurate, as muscles which are stretched on loading, as the kangaroo ankle extensors are during hopping, can develop greater than isometric stresses (^24^, pp. 21–23). However, while incorporating this factor might affect our absolute values, it would affect each individual equally, and thus could not impact the relative patterns found here: that the giant kangaroos all fall within the relative tendon width range of living kangaroos, which are already known to hop. Thus, our conclusions would remain unaffected by this consideration.

Overall, our data suggests that the giant extinct species favour a broader gastrocnemius tendon relative to body size than today’s kangaroos, protecting the tendon against rupture. This aligns with McGowan’s findings of larger ankle extensor tendons in giant kangaroos potentially allowing for hopping in these species^13^. However, the low safety factors of the ankle extensor tendons in today’s large kangaroos are not simply a liability. In stretching the tendons as close to their breaking point as possible, the potential for elastic energy storage is maximised^15,25,26^. The thicker tendons of the giant kangaroos likely could not store and return as much energy as those of today’s large hopping kangaroos^8^. Previous authors have suggested that thicker tendons would limit the capability of sthenurines to hop because they would be unable to recover sufficient elastic energy to make it worthwhile^8^. However, gait choice in tetrapods is complex, and bipedal hopping may have provided an option for rapid short-distance locomotion even if the elastic energy storage associated with long-distance highly-efficient hopping was unavailable, therefore this argument seems insufficient to rule out hopping.

### Broader implications

In fact, hopping with lower energetic efficiency is already seen in today’s smaller hopping species—both smaller macropodiforms and various hopping rodents—whose tendons are too relatively thick to store much elastic potential energy, but who instead use their hopping abilities to navigate difficult terrain and escape predators^27–29^. While a giant kangaroo would of course not jump vertically to several times its own body height in the way that, for example, a jerboa would^28^, the evolution of hopping in these small extant species helps to demonstrate the versatility of the gait, and that it might be valuable to retain even if it is no longer especially energetically efficient. We suspect from evidence such as tooth marks on giant kangaroo bones attributed to *Thylacoleo*^30^ that retaining hopping as a fast gait may have been necessary for evading predators, in at least some species of giant kangaroo. This necessity most likely varied between species, depending on the local ecology, however.

Moving away from a reliance on efficient hopping may also have some benefits, such as alleviating constraints on posture. Some giant species may have been able to sacrifice the ideal crouched hopping stance, and adopt a more upright posture, further reducing the stress during locomotion, as observed in other mammals to compensate for increases in mass^4,20,21^. For example, *Sthenurus stirlingi*, a large sthenurine species, seems to have an astragalus best suited to a more upright limb posture than the smaller members of the group^31^. Other morphological adaptations to a more upright posture have also been noted in the sthenurines, including a dorsally-tipped ischium and very large epipubic bones indicating an upright trunk, as well as the short calcaneum possibly supporting a more obtuse ankle joint angle^8^.

For giant sthenurines and *Protemnodon* species, previous investigations have proposed alternative gaits they may have used instead of hopping. The most-studied group is the Sthenurinae. A variety of anatomical features—including a pelvis which seems to reflect an upright posture, a broad sacrum and a stabilised ankle joint^8^; the morphology of the articular surfaces of the humerus^9,32^ and the astragalus^31^; and cortical thickening in the pedal bones^22^—support an ability to stride bipedally. A fossil sthenurine trackway has also been reported which shows bipedal striding^33^. Meanwhile, a recent study^34^ compares the limb indices of various modern and fossil kangaroos, and finds that the limb indices of both large *Protemnodon* species investigated in this study (*P. anak* and *P. viator*), together with anatomical features such as hooked phalanges (both species) and an elongated neck (*P. anak*), suggest they may have been primarily quadrupedal. Other studies which touch on *Protemnodon* anatomy seem to support this hypothesis^8,9,32,35^. However, the most detailed analysis to date, by Kerr et al.^19^, concludes that *P. anak* and *P. viator* were most likely capable of bipedal hopping, as well as pentapedal slow locomotion, based on features such as the morphology of the proximal tail vertebrae. This study also highlights the likely interspecific variation in *Protemnodon* locomotion, a point that is worth bearing in mind when interpreting the locomotor abilities of all lineages of giant kangaroos. In our own results, however, we find the same ultimate conclusion—that hopping is plausible—for all giant species included in the analysis.

For the remaining group of giant kangaroos, the giant *Macropus* species, no other primary gait besides hopping has yet been proposed. They are consistently found to be more anatomically similar to today’s large hopping kangaroos than the Sthenurines and large *Protemnodon* species are^8,9,22^. This is likely to be at least partly due to phylogenetic constraints—they are more closely related to today’s *Macropus* species, and therefore would be expected to be less morphologically divergent—but the fact remains that they attained large sizes without significantly adapting a body plan specialised for hopping. It has previously been suggested that *Macropus giganteus* underwent within-species ‘dwarfing’, appearing both as a giant Pleistocene species and a modern large kangaroo^36^. If true, this would have many implications for the question of locomotion in giant kangaroos: for example, it might be considered as evidence that one giant kangaroo species, at least, did hop. However, the taxonomy of *Macropus*species is rather uncertain to date (e.g^37^.), and resolving this issue is beyond the scope of the current analysis, so drawing confident conclusions from this purported ‘dwarfing’ is likely unsupportable. In support of the idea that these giant *Macropus* species did hop, this study finds that, as with the sthenurines and *Protemnodon*, both fourth metatarsals and gastrocnemial tendons could have supported hopping. Likewise in support of this idea, the calcanea of the giant *Macropus* species have been found to have extensive cortical thickening, similar to that seen in modern large kangaroos^22^. This is likely an adaptation to resist high forces exerted by the ankle extensor tendons when hopping, potentially suggesting a more active mode of locomotion than used by the sthenurines, which do not show this pattern of cortical thickening^22^. However, giant *Macropus* species do share with the giant sthenurine and *Protemnodon* species included in this study the pattern of a broader, shorter calcaneum relative to today’s large kangaroos (Fig. S4). As previously discussed, this suggests that even if hopping was the primary mode of locomotion used by this group, it may have been less efficient than in the largest extant hoppers.

Overall, nothing in our analyses suggests that it would have been mechanically impossible for any giant kangaroo species included in this study to hop. However, they may not have been as well-adapted for fast, sustained or efficient hopping as their largest living relatives. Instead, incorporating a variety of other gaits into their repertoire may have helped the giant kangaroos to reach sizes and ecological niches unexploited by today’s macropodoids. The diversity of proposed locomotor modes in the giant kangaroos reflects a wider ecological diversity in the kangaroo populations of the Pleistocene than is seen today: for example, there is evidence that the sthenurines were large browsing species^38,39^—a niche not occupied by modern large kangaroos—while other giant species were grazers^40,41^, indicating greater dietary diversity in the past.

## Methods

### Specimens

All species included in this study were macropodiforms; the bone measurement dataset encompassed all extant families and subfamilies of Macropodiformes, and several major extinct lineages (Sthenurinae, Balbaridae, *Protemnodon*, the giant *Macropus* species). 179 specimens were measured in total, across 63 species and 25 genera. Of these, 139 specimens were modern, and 40 fossil. Many specimens had some missing data, and so were not included in all analyses (Table S1). For the analyses shown in the main body of this paper, 134 specimens (94 modern, 40 fossil) were used in total, with 89 specimens (65 modern, 24 fossil) being used to evaluate hypothesis one (Fig. 3), and 46 specimens (30 modern, 16 fossil) used in the final evaluation of hypothesis two (Fig. 5). The remainder are only referred to in the supplementary material. This was necessary as the main analyses, especially of hypothesis two, require a variety of measurements from articulated specimens of the kangaroo pes, which are relatively rare among fossils particularly. Body masses were gathered from the literature (^16,22,36,42–49^ for details, see Table S1). Where possible, the mass of the individual was used, but where this was not available, the mean body mass, corresponding to either the sex of the individual (in strongly dimorphic species), or the species as a whole, was used instead. An attempt was made to extrapolate body mass from calcaneal measurements instead, following Prideaux and Warburton (2023), on the basis that this uses direct evidence from the individual specimens used rather than species means. However, due to the disproportionate shortening and broadening of the calcanea in the giant kangaroos—see later discussion—this produced implausibly low estimates of body mass for all giant kangaroos.

All PCSA data used in this paper is from the previously published paper by McGowan et al.^10^, and as such is not separately published here.

### Morphological data

Articular lengths of key hindlimb bones (the femur, tibia, fourth metatarsal, fourth proximal phalanx, and calcaneum) were collected, as well as antero-posterior and medio-lateral midshaft widths of the fourth metatarsal and width of the calcaneal tuberosity, where available (Fig. 2). Some of these measurements were taken from the literature^16,22^ and private correspondence (*n* = 317, *nspecies* = 65); others were collected for this study by the authors (*n* = 65, *nspecies* = 38). Details of specimens, including specimen numbers, and sources of body masses and bone dimensions can be found in Table S1. For some of these specimens, an additional set of calcaneal dimensions (31 specimens: 11 fossil, 20 modern) were collected to facilitate interpretation of the second hypothesis results (Table S1; Fig. 2). For each specimen, digital callipers were used to measure the width of the calcaneal tuberosity at its widest point, the calcaneal length (taken along the mediolateral centre of the bone), and the mediolateral and dorsoventral widths of the calcaneum, taken halfway along the length of the bone. Where available, the length of the associated fourth metatarsal was also measured. Measurements were taken to the nearest 0.01 mm. For all data collected for this study, see Table S1.

### Ankle moments when hopping

**Fig. 6:**
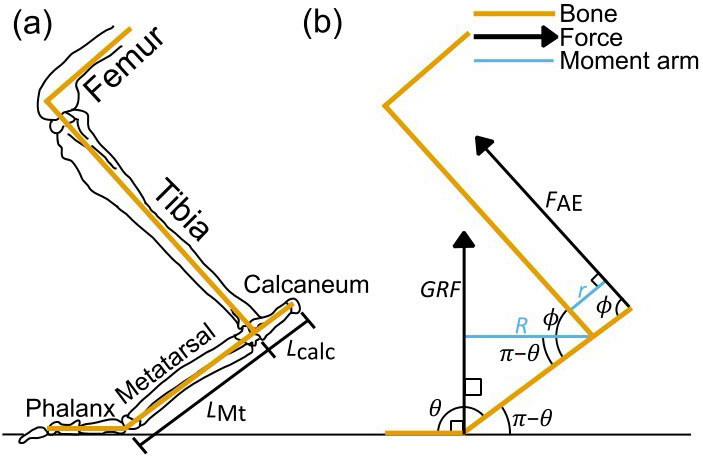
(a) Schematic drawing of the distal hindlimb bones of *Macropus giganteus*, adapted from^25^, with key measured bone lengths labelled, and (b) a free-body diagram illustrating the terms used in the text for forces and angles (black), as well as lever arms (blue). Red indicates the bones themselves. Abbreviations: F_AE_ = force exerted by ankle extensors; GRF = ground reaction force; L_calc_ = length of the calcaneum; L_Mt_ = length of the metatarsal; R = lever arm of GRF; r = lever arm of F_AE_; = metatarsophalangeal joint angle; = ankle joint angle. Hindlimb image by MJ, based on^10,25,59^.

To test our hypotheses, we first needed to estimate the moments experienced around the ankle joint of each specimen when hopping (Fig. 6). Kangaroo joint angles can differ among species and with hopping speed^14^. However, limited data are available, and while joint angles do vary, this variation is relatively small, as demonstrated by the constant effective mechanical advantage at the ankle joint among species^25^, and at different speeds within a species^12^. Thus, the joint angles at midstance to the nearest 5 degrees for *Notamacropus eugenii* (see Fig. 3 of^50^), are here taken as representative for all species. This species was used as it provides the best currently available data on joint angles throughout a hopping cycle, and as a midsized wallaby, it is a reasonable choice for a representative species. “Midstance” was defined as the point of peak ankle flexion during the stance phase. The mean angle derived from three stance phases gave a metatarsophalangeal joint angle of 1.95 radians (112°), and an ankle joint angle of 1.60 radians (92°). As a recent study found that joint angles can vary somewhat by body mass^51^, a sensitivity analysis was also performed, varying each of these joint angles by 10% towards a more or less crouched posture (Fig. S3) to see if this affected the final conclusions. The conclusions of neither hypothesis were affected by this change, so we retain these assumed joint angles for the remainder of the study. From the metatarsophalangeal joint angle (=1.95), and the length of the fourth metatarsal (*L*_Mt_), the moment arm (*R*) of the ground reaction force at midstance was calculated:

1 $$R = L_{{Mt}} \cos (\pi - \theta )$$

The peak ground reaction force (*GRF*) acting on each individual hindlimb was assumed to be three times the weight (3 *mg*) of the animal, occurring at midstance and being oriented vertically^52^. Although a peak ground reaction force of 5 *mg* has been recorded in red kangaroos^14^, this seems to be a value for the whole animal (both hindlimbs), rather than for the hindlimbs considered individually, which would imply that each limb experienced ~ 2.5 *mg* of force. Thus, 3 *mg* was considered a conservative estimate for hopping animals, and this value was used here. The vertical orientation of GRF is in line with the results of a recent study which measured hopping kangaroos on a force plate^51^. From the peak GRF and the GRF moment arm *R*, the moment at the ankle joint was calculated as:

2 $$M_{{{\mathrm{GRF}}}} = GRF \cdot R$$

GRF is here assumed to act at the metatarsophalangeal (MTP) joint. A sensitivity analysis was also run, comparing the results shown here to those found if GRF was assumed to act at the midpoint of the phalanges, assuming that the first phalanx represented 42% of the total phalanx length (a mean value derived from data provided by Christine Janis, pers. comm.). The results of this sensitivity analysis (Fig. S2) are consistent with our findings when GRF is assumed to act at the MTP joint, with fossil individuals falling within the safety factor ranges of modern kangaroos. However, it also predicts a safety factor of < 1 for many modern kangaroos, including those for which GRF values close to our assumed value have been recorded (see figure below). We know this to be inaccurate, as these species do hop without fracturing their metatarsals. Therefore, we conclude that our original assumption of GRF acting at the MTP joint is more likely to produce an accurate model of hopping abilities among extinct species in this case. Thus, we proceed with this assumption for the remainder of the study.

### Hypothesis 1: metatarsal safety factors

For those specimens where the antero-posterior (AP) and medio-lateral (ML) diameters of the fourth metatarsal were known (*n* = 89), the second moment of area at the midshaft (*I*) was predicted as follows^53^:

3 $$I = (\pi\cdot{r_{ml}}\cdot{r_{ap}} ^{3} )/4$$

Where *r*_ml_ is the mediolateral radius, and *r*_ap_ is the anteroposterior radius. Then, the bending moment of the GRF at the midshaft (*M*_mid_) was calculated:

4 $$M_{{mid}} = GRF \cdot 0.5L_{{Mt}} \cdot \cos (\pi - \theta )$$

Next, peak stress at the midshaft () was calculated based on these values for M_mid_, = *r*_ap_, and *I* (from^53^, p. 16]):

5 $$\sigma = (M_{{mid}} \cdot r_{{ap}}) /I$$

The safety factor of the metatarsal at peak stress was calculated by dividing the bending failure strength of mammalian bone by the peak stress recovered above. The failure strength of mammalian bone varies somewhat across species and bone type; for the sake of this study, we approximate it as 200 MPa in kangaroos. This value is the mean found for larger mammals in a study by Biewener^21^, and is within the range of values found for the kangaroo rat, the most comparable species in terms of locomotion included in this study.

### Hypothesis 2 preparation: ankle extensor muscle physiological Cross-sectional areas (PCSAs)

To test the second hypothesis, we must estimate the force the ankle extensor muscles exert on their tendons, in order to estimate the requisite tendon cross-sectional area, and thus width, to resist this force. We employ two different methods to make this estimate.

The first method predicts ankle extensor muscle PCSAs in the giant kangaroos from extrapolated allometric scaling patterns. The measured PCSAs of ankle extensor muscles for a variety of modern macropodoids were collected from the literature (^10^, provided by Craig McGowan, Pers. Comm.), including values for the gastrocnemius (GAS), plantaris (PL), and flexor digitorum longus (FDL). The PCSA values of these three muscles were summed to produce a total ankle extensor muscle PCSA. Linear ordinary least squares regressions were then performed on the log_10_-transformed PCSA and body mass data for three datasets: (1) the PCSAs estimated from ankle moments; (2) the summed measured ankle extensor PCSAs; and (3) the measured gastrocnemius PCSAs (Fig. 4). It is worth noting that the FDL possesses a reduced lever arm, relative to the other ankle extensor muscles, as it passes closer to the rotational centre of the ankle joint, meaning that it contributes less to the effective PCSA required to balance ground reaction forces at the ankle. Since we do not have specific data on the moment arm of the FDL, we disregard this muscle and the plantaris in the subsequent calculations of ankle extensor tendon width, in favour focussing on the gastrocnemius muscle. The gastrocnemius tendon is also the only one which inserts directly on the calcaneal tuberosity, meaning that this is the tendon which determines if adequate insertion area is available on the calcaneal tuberosity.

This method does rely on extrapolation beyond the mass range of living species, which, as previously discussed, is not ideal, and means that these particular estimates are subject to the same issues mentioned for previous studies. However, there are no available osteological indicators of the size of the extensor muscles in giant kangaroos. The scaling relationships for ankle extensor muscles among modern kangaroos are hyper-allometric, with PCSA ∝ *M*_b_ (Fig. 4), whereas based on isometry, the only other option we have for estimating PCSA from body mass, we would expect PCSA ∝ *M*_b_^2/3^. Therefore, it is likely that if this extrapolation from living species is inaccurate, it is an overestimate of the PCSA available for the extinct species, if they did not hop, and is thus a conservative estimate relative to our hypothesis.

Our second method, however, does not rely on allometric extrapolation at all, instead using our estimate of peak ground reaction force (GRF) and measured bone lengths to calculate the minimum force the ankle extensor muscles must produce to resist GRF. It thus avoids the problems which come with allometric extrapolation. For this method, the amount of force the ankle extensor muscle-tendon units (MTUs) were required to produce (*F*_AE_) to balance the moment of the GRF at the ankle joint was calculated as:

6 $$F_{{{\mathrm{AE}}}} = M_{{{\mathrm{GRF}}}} /r$$

where *r* is the moment arm of the ankle extensor MTUs. To find this moment arm, both the length of the calcaneum and the angle between the calcaneum and the ankle extensor MTUs needed to be known. The length of the calcaneum in each case was already in our measured dataset. Meanwhile, the line of action of the MTUs was assumed to run parallel to the tibia, and the calcaneum parallel to the metatarsal, meaning that the angle between the two is the same as the ankle joint angle ($\phi$) (Fig. 6). Thus, *r* was calculated as:

7 $$r = L_{{{\mathrm{calc}}}} \sin \phi$$

where *L*_calc_ is the length of the calcaneum.

From the calculated ankle extensor force, the required total ankle extensor muscle PCSA (in m^2^) was calculated by dividing *F*_AE_ by 3,000,000—since the maximal isometric stress of the muscles was assumed to be 0.3 MPa, following McGowan et al.^10^ for consistency with prior studies. This calculation provides a measure of the minimum ankle extensor muscle PCSA required to balance the moments involved in hopping.

### Hypothesis 2: ankle extensor tendon width

To test our second hypothesis, the muscle PCSAs calculated in the previous section were used to predict the minimum tendon diameter required to maintain a tendon safety factor above one when hopping. From the PCSA of a muscle, the theoretical maximum force can be calculated; from this the minimum cross-sectional area (CSA), and then the tendon diameter needed to withstand this force can be derived. To accommodate hopping without tendon rupture, the calcaneal tuberosity width, a proxy for the maximum possible diameter of the tendon, must exceed this minimum required tendon diameter.

Three sets of predicted tendon diameters were created. The first was derived from the moment-based estimation of the ankle extensor muscle PCSA created in the section above, and represents the absolute minimum tendon size required to prevent rupture during hopping. The second was derived from the gastrocnemius PCSA regression equation calculated from measured PCSAs in modern kangaroos^10^ in the section above, and represents the tendon size if we assume similar muscle scaling to living species. The PCSA estimates from the first two methods were used to predict minimum tendon CSA as follows:

The maximum stress experienced by a tendon (*σ*_*t*_) is equal to the maximum isometric stress which can be exerted by the muscle—assumed to be 0.3 MPa—multiplied by the ratio of muscle physiological cross-sectional area (*A*_*m*_) to tendon cross-sectional area (*A*_*t*_)^10^:

8 $$\sigma _{t} = 0.30(A_{m} /A_{t} )$$

The safety factor of the tendon can be calculated by dividing the failure strength of the tendon—assumed to be 100 MPa, once again following the methods of McGowan et al. (2008)—by *σ*_*t*_.

9 $$SF_{t} = 100/\sigma _{t}$$

If we assume a safety factor of 1, then using the above equations, we find that:

10 $$A_{m} /A_{t} = 333.3$$

A safety factor of one is lower than would be acceptable in real life, given that a safety factor of < 1 would indicate tendon rupture. However, this value is used here to represent the absolute lower limit of tendon safety factors. Equation 10 was used to calculate the minimum tendon cross-sectional area (CSA) for all modern and fossil individuals where calcaneal measurements and *M*_b_ values were available, based on the two muscle PCSAs described above.

A third estimate of tendon diameter was derived from an existing regression equation for tendon CSA against mass^10^. While this approach relies entirely upon extrapolation from modern data, it was included for comparison to the previous two approaches, and allows us to assess the sensitivity of our conclusions to changing the method for estimating tendon CSA in extinct species.

The gastrocnemius tendon diameter was calculated from all three sets of tendon CSA predictions. To do this, a reasonable estimate of tendon ellipticity at insertion is required. To our knowledge there are no published data on the major vs. minor axis dimensions of kangaroo hindlimb tendons. The wider literature on mammal gastrocnemius tendons is likewise limited. Peterson et al.^54^ state that, across mammals, the anteroposterior and mediolateral widths of the tendon “are rarely very different because the tendon is quite round at its thinnest point”. This is not necessarily reflective of the cross-sectional shape at insertion, however, and they do not provide raw data, so the details cannot be judged. However, Obst et al.^55^ find that, in humans (who may be more comparable to kangaroos than to most mammals, as kangaroos are bipedal), the Achilles tendon is highly elliptical at insertion on the calcaneum. Raw data for this study is likewise not available, but based on values attained from digitising Fig. 3 using WebPlotDigitizer^56^, the major axis is 4.80 times greater than the minor axis at rest, and 4.94 times greater at maximal contraction. With this degree of ellipticity assumed, our projected required tendon widths are increased by a factor of 2.24, relative to a circular cross-section. If the resulting minimum tendon diameter exceeds the measured calcaneal width, then tendon rupture would be likely during hopping locomotion and it can be ruled infeasible.

The resulting tendon width predictions were compared to each other, and to the measured widths of the calcaneal tuberosities for the same species, to see if the tendons would fit the calcanea observed in the fossil record. We do not suggest that there is a predictable relationship between calcaneum width and tendon size, as the tendon may not insert on the entire width of the calcaneal tuberosity. However, we calculate the ratio of the three sets of predicted tendon sizes to measured calcaneal width for modern and fossil kangaroos, to see whether there is any evidence that the fossil specimens were closer to being unable to accommodate the tendons required for hopping than any of their living relatives.

All statistics performed in this study were linear least-squares regressions performed in base R v.4.4.1^57^, with plots produced using the package ggplot2^58^.

## Supplementary Information

Below is the link to the electronic supplementary material.

Supplementary Material 1

Supplementary Material 2

## Data Availability

Data is provided within the manuscript or supplementary information files.
