# Supplementary material for: Biomechanical limits of hopping in the hindlimbs of giant extinct kangaroos
Source: Sci Rep. 2026 Jan 22;16:1309. doi: 10.1038/s41598-025-29939-7 (PMC12828012; doi:10.1038/s41598-025-29939-7)
Supplement: Supplementary file 1 — Supplementary Material 1 [file 41598_2025_29939_MOESM1_ESM.pdf]

The intra-hindlimb proportions of Macropodiformes

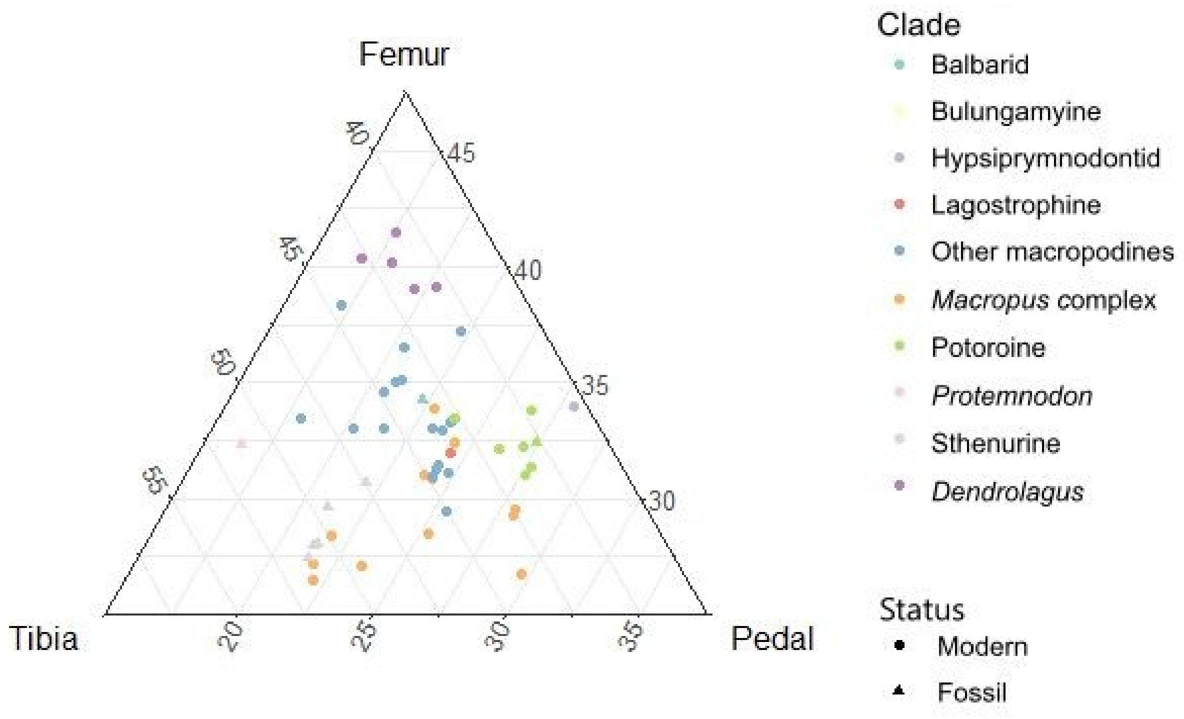

Fig. S1 Ternary diagram of the length of the tibia, femur, and pedal bones (fourth metatarsal + associated proximal phalanx) of macropods. Each value is the length of the segment as a proportion of the entire leg. Each point is the species mean. The colours of the points denote clades within the Macropodiformes, while shape denotes whether a species is extant or extinct. *n* = 116 specimens, 50 species means.

Methods

A ternary diagram using species means of bone lengths was produced in R, showing the relative proportions of the femur, tibia, and foot bones (metatarsal + proximal phalanx) for each species.

Results

The ternary diagram (Fig. S1) shows that relative hindlimb proportions among macropodiforms generally cluster by taxon. The tree-kangaroos are the most distinct group, with short tibiae and feet, reflecting their unique mode of locomotion among extant macropodiforms. Among the extinct groups, the balbarid *Ganawamaya gillespieae* clusters in the centre of the space occupied by extant macropodiforms, closest to the non-*Macropus*-complex Macropodinae. *Protemnodon* has short feet, much like the tree-kangaroos, but has a similarly elongated tibia to the *Macropus* complex. Finally, the Sthenurinae occupy the leftmost side of the range of the *Macropus* complex, indicating similar general hindlimb proportions to that group, although they tend to have somewhat shorter feet than many members of the *Macropus* complex.

Sensitivity analysis of GRF position

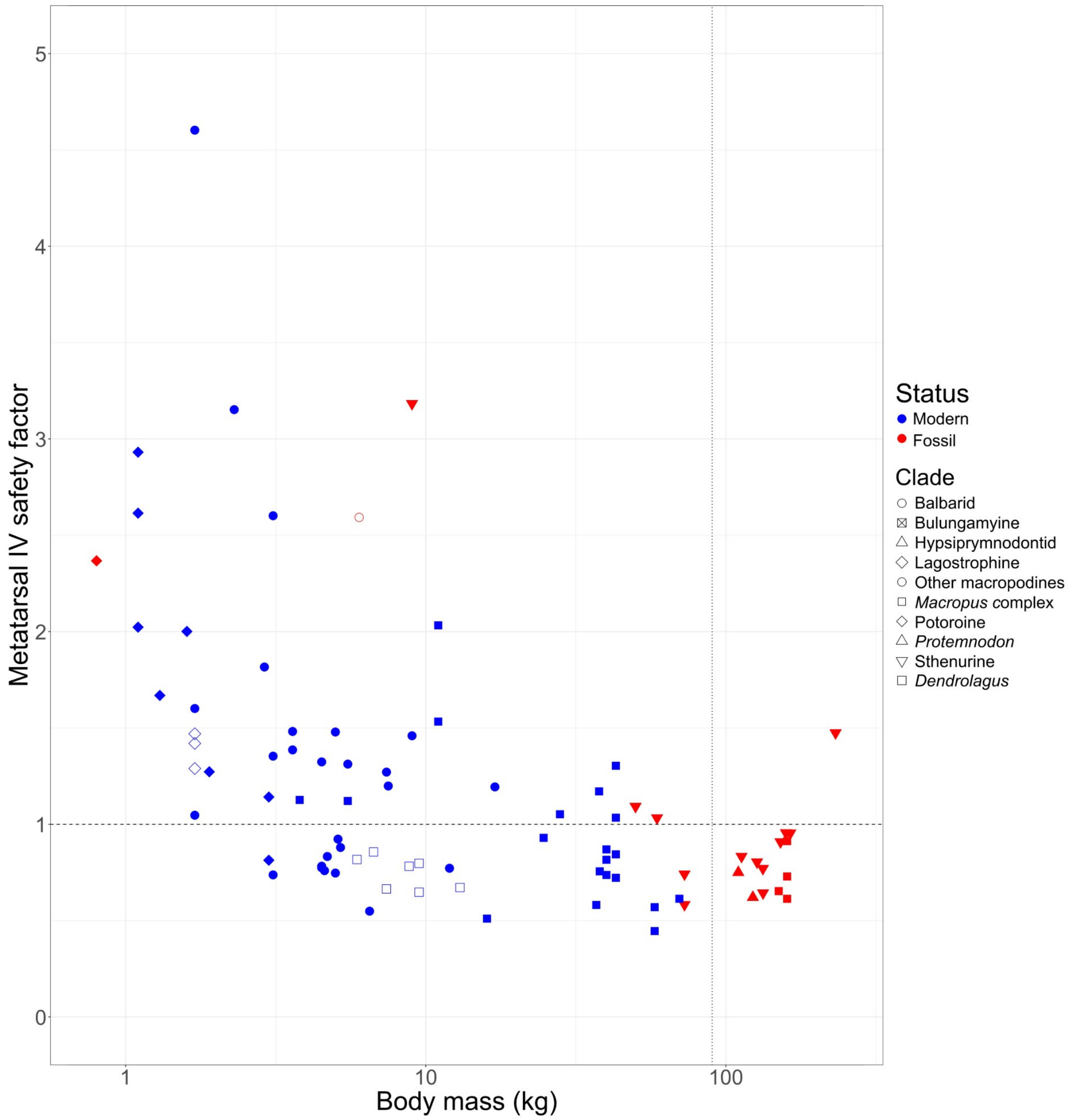

Fig. S2: Scatterplot of the predicted safety factor of metatarsal IV at midstance when hopping, against log-transformed body mass, **assuming GRF acts halfway along the phalanges, where the first phalanx makes up 42% of the total length of the phalanges**. The shapes of the points denote clades within the Macropodiformes, while colour denotes whether a species is extant or extinct. The horizontal dashed line indicates a safety factor of one, below which the bone would be expected to risk fracture. The vertical dotted line indicates the mass of the largest extant kangaroos. n = 89 individuals.

Sensitivity analysis of joint angles

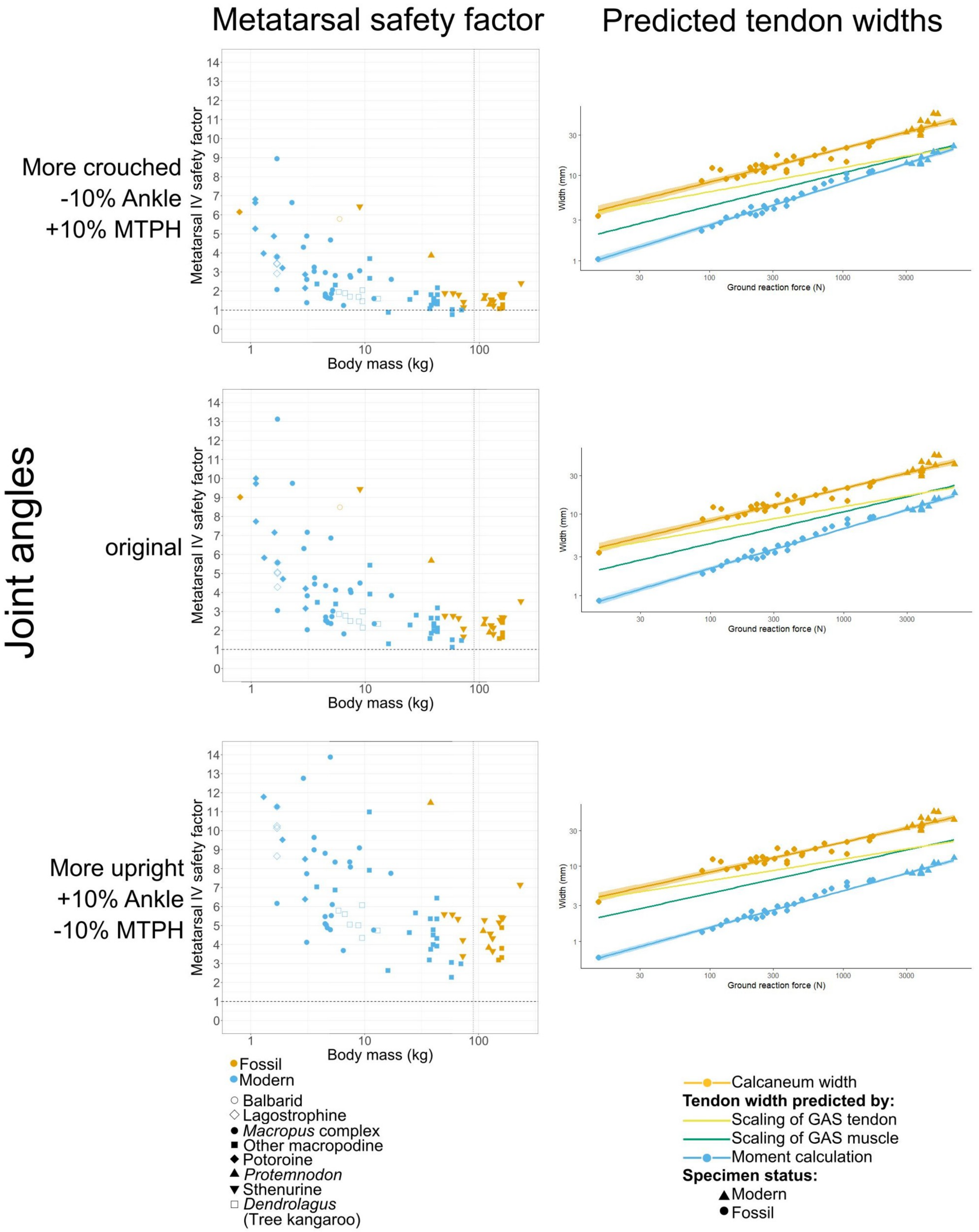

Fig. S3: Sensitivity analysis of the results to joint angles. Graphs show the impact of increasing/decreasing joint angles by 10% on Fig. 2 (metatarsal safety factors, hypothesis 1) and Fig. 4 (predicted width of the gastrocnemius tendon, hypothesis 2) from the main text.

The relationship between calcaneum dimension ratios and body mass

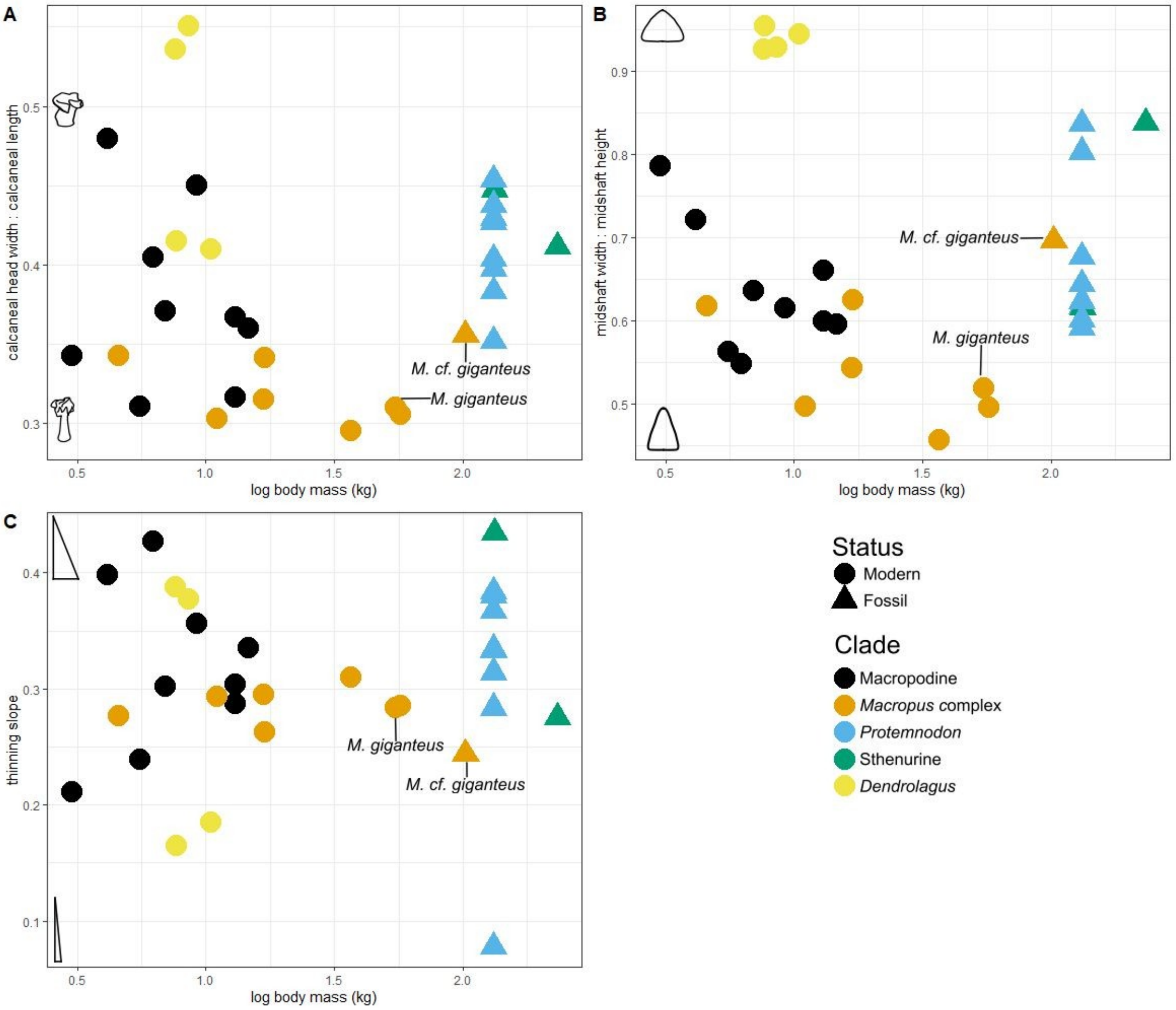

Fig. S4 Calcaneum dimension ratios against log-transformed body mass. (a) Calcaneal head width to calcaneal length; (b) midshaft width to midshaft height; (c) slope of calcaneal head width to midshaft width ("thinning slope"). Points are coloured by clade. n = 31.

Methods

Three indices were calculated for the calcaneal dataset collected in the NMV (Museums Victoria), and plotted against mass. These were: ratio of calcaneal head width to total calcaneal length; ratio of mediolateral width at midshaft to dorsoventral width at midshaft; and the slope of mediolateral width from the calcaneal head to the midshaft (calculated as the difference between the two mediolateral widths, divided by half the total calcaneal length). Together, these indices help us to broadly characterise calcaneal morphology, and to see if there are any adaptations of the insertion point of the gastrocnemius tendon which should be taken into consideration when interpreting results from the main study.

Results

The calcanea of giant extinct kangaroo species were shorter relative to calcaneal head width (Fig. S4a), and less mediolaterally compressed at the midshaft (Fig. S4b) than in smaller extant species. This is a departure from a general pattern among extant species of longer, narrower calcanea with increasing size. The third index, the rate at which the calcaneum narrows from the tuber calcanei to the midshaft (Fig. S4c), seems less informative, with no clear trend over size or between clade groups (including in the extinct species).

Discussion

While the thinning slope (Fig. S4c) seems relatively uninformative, the other two indices both indicate a departure from allometric patterns in the calcanea of extinct giant kangaroos. In living species, the calcanea grow relatively longer and mediolaterally narrower with size. Meanwhile, the relative dimensions of the extinct species' calcanea resemble those of small macropodines, and even some tree kangaroos, far more than large extant kangaroos. The calcaneal morphology of large extant kangaroos is surely strongly influenced by the high forces exerted on the tuber calcanei by the gastrocnemius tendon when hopping. The large kangaroos face a trade-off in their ankle extensor tendon dimensions. A narrower tendon is able to store and return more elastic energy, but is less able to withstand high forces (McGowan et al., 2008; Bennett and Taylor, 1995). Thus, a kangaroo adapted to optimise efficient hopping would be expected to have a tendon (and corresponding tuber calcanei) with the narrowest width possible without risking tendon rupture. A relatively longer calcaneum may also be advantageous, providing the gastrocnemius tendon with a greater in-lever, and resulting in the tendon being stretched over a longer distance at the mid-stance of a hop, increasing elastic strain energy storage. The changing dimensions of calcanea from small to large Macropodinae seem to reflect this adaptation to maximum efficiency, with increased relative length and a more narrow shaft, and no particularly great flaring of the shaft towards the tuber calcanei (Fig. S4). The fact that the sthenurines and *Protemnodon* depart from this, seemingly more than would be demanded by allometry, and adopt proportions more comparable to small macropodines, may suggest a shift away from prioritising efficiency in hopping.

Macropus giganteus is a species which may have become smaller after the Pleistocene, rather than becoming extinct (Helgen et al., 2006). Today, the species has a body mass on a par with other large extant kangaroos. This assertion depends, however, on the accuracy of the taxonomy of fossil *Macropus* species, which is currently debated. Our calcaneal dataset includes one purported *M. giganteus* specimen from the Pleistocene, and one modern specimen. This Pleistocene *Macropus* cf. *M. giganteus* specimen follows the pattern of other giant species, possessing a relatively shorter, broader calcaneum. The import of this, however, depends on whether this is indeed *M. giganteus*, a question beyond the scope of this study.

Wagstaffe et al. (2022) studied the second moments of area and cortical bone distribution of sthenurine and macropodine calcanea. They found that sthenurine calcanea are less resistant to bending than macropodine calcanea, and suggest that the broader heads of sthenurine calcanea reflect a movement away from optimising the elastic energy storage capacity of the gastrocnemius tendon. Their findings do suggest that sthenurine species were not optimally adapted for hopping, but also seem to indicate that this correlates more with phylogeny than body mass, as the paper compares sthenurines and macropodines of similar body masses, and finds these differences throughout. The largest specimen they studied, *Macropus* cf. *M. titan* (estimated at 176 kg), retained a strengthened calcaneum with high resistance to bending, and the authors predicted that this species would still have hopped, despite its size. Unfortunately, no *M. titan* specimens are included in our own dataset. However, the single giant extinct macropodine we did measure, *Macropus giganteus*, shows deviations in shape from the smaller macropodines, closer to the ranges of the even larger sthenurine and *Protemnodon* specimens. So, in contrast to the findings of Wagstaffe et al. (2022), we do find that giant macropodines may show physical adaptations which suggest a move away from optimisation for hopping locomotion.

Supplementary Tables

Table S1: All specimens in the study, their limb bone lengths and body mass measurements. Mass/length source abbreviations: ADW = Animal Diversity Web (University of Michigan Museum of Zoology, n.d.), Av_Oth = Average (mean) of other estimates for the same species in the dataset, B = Butler et al. (2017), CJ = Christine Janis (Pers. Comms), CJIJ = Photograph with scale provided by Christine Janis through personal correspondence, measurements taken in ImageJ 1.53K, D = Dawson (2006), EC = Dawson (1995), GB = Green-Barber et al. (2018), H = Helgen et al. (2006), MJIJ = Photograph with scale taken by Megan Jones, measurements taken in ImageJ 1.53K, NMV_lab = Mass marked on the specimen label, Museums Victoria, NMV_MJ = NMV specimens measured with callipers by Megan Jones, RB = CT scans provided by Roger Benson, via the project TEMPO Mammals (Project ID 00000C428) on MorphoSource.org (Duke university). Funding for this collection was provided by the European Research Council (ERC) starting grant TEMPO (ERC-2015-STG-677774). The CT scans were segmented and measurements taken in Avizo, RR = Rose and Rose (2018), Sch = Schulz (1997), SD = Silva and Downing (1995), Sim_M_g = Similar size to *Macropus giganteus*, Sim_P_g = Similar size to *Protemnodon gilli*, Th = Thornton et al (2021), Th_B = Thornton et al. (2021), referencing Bauschulte (1972), Th_D = Thornton et al. (2021), referencing Doube (2018), Th_Ja = Thornton et al. (2021), referencing Janis et al. (2014), Th_Jo = Thornton et al. (2021), referencing Johnson (1982), Th_K = Thornton et al. (2021), referencing Kear (2008), Th_McG = Thornton et al. (2021), referencing McGowan et al. (2008), Wa = Wagstaffe et al. (2022), WaIJ = Photograph with scale taken from Wagstaffe et al. (2022), measurements taken in ImageJ 1.53K, WM = Wilson and Mittermeier (2015). Institution codes: AM = Australian Museum (Sydney, NSW, Australia), AMNH = American Museum of Natural History (New York, USA), FMNH = Field Museum of Natural History (Chicago, IL, USA), NHMUK = Natural History Museum (London, UK), NMV = Museums Victoria (Melbourne, VIC, Australia), NTM = Northern Territories Museum and Art Gallery (Alice Springs, NT, Australia), QM = Queensland Museum (Brisbane, QLD, Australia), SAM = South Australian Museum (Adelaide, SA, Australia), UCMP = University of California Museum of Palaeontology (Berkeley, CA, USA), UMZC = University of Cambridge Museum of Zoology (Cambridge, UK), WAM = Western Australian Museum (Perth, WA, Australia)

References

- Bennett, M.B. & Taylor, G.C. Scaling of elastic strain energy in kangaroos and the benefits of being big, *Nature*, **378**, 56–59 (1995).
- Butler, K., Travouillon, J., Price, G.J., Archer, M. & Hand, J.S. Species abundance, richness and body size evolution of kangaroos (Marsupialia: Macropodiformes) throughout the Oligo-Miocene of Australia, *Palaeogeography, Palaeoclimatology, Palaeoecology*, **487**, 25–36 (2017).
- Dawson, L. An ecophysiological approach to the extinction of large marsupial herbivores in middle and late Pleistocene Australia. *Alcheringa Australas. J. Palaeontol.* **30**, 89–114 (2006).
- Green-Barber, J. M. & Old, J. M. Town roo, country roo: a comparison of behaviour in eastern grey kangaroos *Macropus giganteus* in developed and natural landscapes. *Aust. Zool.* **39**, 520–533 (2018).
- Helgen, K.M., Wells, R.T., Kear, B.P., Gerdtz, W.R. & Flannery, T.F. Ecological and evolutionary significance of sizes of giant extinct kangaroos, *Australian Journal of Zoology*, **54**, 293–303 (2006).
- Janis, C. M., Buttrill, K. & Figueirido, B. Locomotion in extinct giant kangaroos: were Sthenurines hop-less monsters? *PLoS ONE* **9**, e109888 (2014).
- McGowan, C. P., Skinner, J. & Biewener, A. A. Hind limb scaling of kangaroos and wallabies (superfamily Macropodoidea): implications for hopping performance, safety factor and elastic savings. *J. Anat.* **212**, 153–163 (2008).
- Rose, R. W. & Rose, R. K. *Thylogale billardierii* (Diprotodontia: Macropodidae). *Mamm. Species* **50**, 100–108 (2018).

- Schulz, M. The Diet of the Powerful Owl *Ninox strenua* in the Rockhampton Area. *Emu - Austral Ornithol.* **97**, 326–329 (1997).
- Silva, M. & Downing, J. A. *CRC Handbook of Mammalian Body Masses*. (CRC Press, Boca Raton, 1995).
- Thornton, L. H., Dick, T. J. M., Bennett, M. B. & Clemente, C. J. Understanding Australia's unique hopping species: a comparative review of the musculoskeletal system and locomotor biomechanics in Macropodoidea. *Aust. J. Zool.* **69**, 136–157 (2022).
- University of Michigan Museum of Zoology. ADW: Home. https://animaldiversity.org/. Accessed: 28/08/2024
- Wagstaffe, A. Y., O'Driscoll, A. M., Kunz, C. J., Rayfield, E. J. & Janis, C. M. Divergent locomotor evolution in "giant" kangaroos: Evidence from foot bone bending resistances and microanatomy. *J. Morphol.* **283**, 313–332 (2022).
- Wilson, D.E. & Mittermeier, R.A. *Handbook of the Mammals of the World*. (Lynx, Barcelona, 2015).
